# *Cymbopogon citratus* and Citral Overcome Doxorubicin Resistance in Cancer Cells via Modulating the Drug’s Metabolism, Toxicity, and Multidrug Transporters

**DOI:** 10.3390/molecules28083415

**Published:** 2023-04-12

**Authors:** Mohammed Hasan Mukhtar, Mahmoud Zaki El-Readi, Mohamed E. Elzubier, Sameer H. Fatani, Bassem Refaat, Usama Shaheen, Elshiekh Babiker Adam Khidir, Hesham Hamada Taha, Safaa Yehia Eid

**Affiliations:** 1 Department of Biochemistry, Faculty of Medicine, Umm Al-Qura University, Al-Abdeyah, Makkah 24381, Saudi Arabia; 2 Biochemistry and Molecular Biology Department, Faculty of Pharmacy, Al-Azhar University, Assuit 71524, Egypt; 3 Laboratory Medicine Department, Faculty of Applied Medical Sciences, Umm Al-Qura University, Al-Abdeyah, Makkah 24381, Saudi Arabiaebkhidir@uqu.edu.sa (E.B.A.K.); 4 Department of Pharmacognosy, Faculty of Pharmacy, Al-Azhar University, Cairo 11829, Egypt

**Keywords:** *Cymbopogon citratus*, essential oil, lemon grass, multidrug resistance, doxorubicin, citral, GC-MS

## Abstract

Multidrug resistance (MDR) is the major complex mechanism that causes the failure of chemotherapy, especially with drugs of natural origin such as doxorubicin (DOX). Intracellular drug accumulation and detoxification are also involved in cancer resistance by reducing the susceptibility of cancer cells to death. This research aims to identify the volatile composition of *Cymbopogon citratus* (lemon grass; LG) essential oil and compare the ability of LG and its major compound, citral, to modulate MDR in resistant cell lines. The composition of LG essential oil was identified using gas chromatography mass spectrometry (GC-MS). In addition, a comparison of the modulatory effects of LG and citral, performed on breast (MCF-7/ADR), hepatic (HepG-2/ADR), and ovarian (SKOV-3/ADR) MDR cell lines, were compared to their parent sensitive cells using the MTT assay, ABC transporter function assays, and RT-PCR. Oxygenated monoterpenes (53.69%), sesquiterpene hydrocarbons (19.19%), and oxygenated sesquiterpenes (13.79%) made up the yield of LG essential oil. α-citral (18.50%), β-citral (10.15%), geranyl acetate (9.65%), ylangene (5.70), δ-elemene (5.38%), and eugenol (4.77) represent the major constituents of LG oil. LG and citral (20 μg/mL) synergistically increased DOX cytotoxicity and lowered DOX dosage by >3-fold and >1.5-fold, respectively. These combinations showed synergism in the isobologram and CI < 1. DOX accumulation or reversal experiment confirmed that LG and citral modulated the efflux pump function. Both substances significantly increased DOX accumulation in resistant cells compared to untreated cells and verapamil (the positive control). RT-PCR confirmed that LG and citral targeted metabolic molecules in resistant cells and significantly downregulated PXR, CYP3A4, GST, MDR1, MRP1, and PCRP genes. Our results suggest a novel dietary and therapeutic strategy combining LG and citral with DOX to overcome multidrug resistance in cancer cells. However, these results should be confirmed by additional animal experiments before being used in human clinical trials.

## 1. Introduction

According to the WHO, cancer is a leading cause of death worldwide, killing 10 million people in 2020 [1]. Cancer mortality is expected to rise 45% between 2008 and 2030, exacerbating this situation [1]. The most frequent cancers are breast, lung, colorectal, hepatic, ovarian, prostate, skin, and stomach. Due to a lack of therapeutic specificity, traditional chemotherapies, radiotherapies, and surgeries have many involuntary adverse effects and are not advised for long-term use [2].

Multidrug resistance (MDR) is the leading cause of chemotherapy ineffectiveness in cancer management [3]. It is a multifactorial occurrence resulting from several changes in cancer cells. It may be caused by enhanced drug efflux due to the activation of ATP-binding cassette (ABC) transporters, including multi-resistance protein 1 (MRP1/ABCC1), breast cancer-resistant protein (BCRP/ABCG2), and P-glycoprotein (P-gp/ABCB1/MDR1), stimulation of drug metabolism (Phase 1 mainly CYP3A4 and Phase 2 mainly GST), alteration of drug targeting, inhibition of apoptosis, and modulation of cell cycle checkpoints [4,5].

During the past three decades, cancer biologists have demonstrated a growing interest in the therapeutic utilization of products from natural origins as adjuvant therapy to improve chemotherapy efficacy, enhance the lifestyle of cancer patients, and increase their survival rate [6]. Moreover, a wide array of studies has shown that plant secondary metabolites may iterate cancer cells’ sensitivity to chemotherapeutic drugs [6,7,8,9,10]. Identifying and characterizing inhibitors and modulators of metabolic phase molecules, including Phase 0: efflux pump MDR1, Phase 1: CYP3A4, Phase 2: GST, and Phase 3: MRP1, is one of the important and useful approaches for overcoming MDR. Owing to the importance of metabolic phases in MDR, we have performed comprehensive research to identify natural MDR modulators [8,11,12,13].

The genus Cymbopogon belongs to the family *Poaceae* and is represented in the flora of Saudi Arabia by two species; *Cymbopogon commutatus* and *Cymbopogon schoenanthus* [14]. *Cymbopogon citratus* is commonly cultivated in home and street gardens as a culinary and medicinal aromatic herb because of its scent of strong lemon-like odor. The fresh herb is used in salad and traditional recipes and consumed as a substitute for other tea drinks due to its pleasant flavor [15]. Its traditional name in Saudi Arabia is Izkhir, Athkhar, camel grass or Otrah, and is used in the folk medicine of Saudi Arabia for the treatment of gastrointestinal ailments, insomnia, fever, rheumatism antipyretic, anti-malarial, and anti-helminthic [16], renal antispasmodic [17], and sedative [18] as well as a diuretic to prevent the formation of kidney stones [19,20]. *Cymbopogon citratus* (lemon grass) is this genus’s best-known commercially important volatile oil [21]. Recent biological activities that have been reported for this herb include the prevention of the common cold, flu, cough, high blood pressure, high cholesterol, colorectal cancer, anxiety, toothache, sore throat, antiseptic, insecticidal, antimicrobial, mosquito repellent, anti-inflammatory, anti-mutagenic, cytotoxic, anti-diabetic, antioxidant, and free radical scavenging activities [22,23,24,25]. Phytochemical studies showed that it contains phenols, flavonoids, tannins, saponins, anthraquinones, and alkaloids. The essential oil constituents include aldehydes, alcohols, esters, and other terpenoids [26,27,28]. The essential oil’s chemical makeup shifts based on the plant’s country of origin. Citral, a combination of the geranial (α-citral) and neral (β-citral) isomers, is always the most abundant component. The major terpenoid constituents include citronellol, citronellal, limonene, linalool, nerol, and p-menthane derivatives [26,27,28]. The minor constituents as borneol, myrcene, geraniol, L-linalool, nerol, geranyl acetate, α-terpineol, and elemicin are also present [26,27,28].

Due to the lack of information regarding the LG cultivated in Saudi Arabia as well as its importance in traditional Saudi medicine, the purpose of this study was to investigate the phytochemical constituents of its essential oil and compare the modulatory activity on MDR of LG and citral via the enhancement of doxorubicin cytotoxicity against MDR cells by inhibiting the function and expression of efflux pump and metabolic enzymes.

## 2. Results

### 2.1. GC/MS Identification of Active Constituent of LG

**Figure 1:**
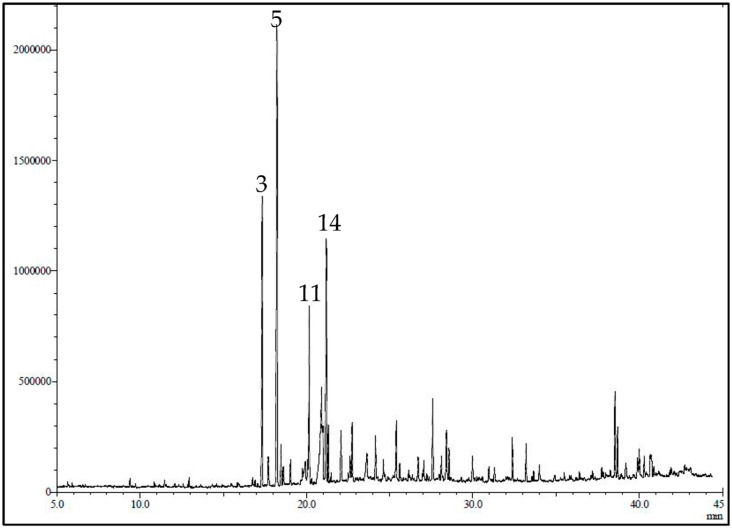
GC-MS profile/chromatogram of the volatile constituents identified in *C. citrates* essential oil. The *y*-axis is the relative abundance of the total ion current (TIC) and the *x*-axis is the retention time (min).

The GC/MS chromatogram of LG is presented in Figure 1. The essential oil yield was 1.2% of the fresh plant material consisting mainly of oxygenated monoterpenes (53.69%), sesquiterpenes hydrocarbons (19.19%), and oxygenated sesquiterpenes (13.79%). Using GC-MS and GC/FID, 36 volatile constituents were characterized and quantified in the essential oil of the leaves of *C. citrates,* representing 95.29% of the total oil composition (Table 1). In the current work, α-citral (18.50%), β-citral (10.15%), geranyl acetate (9.65%), ylangene (5.70), δ-elemene (5.38%), and eugenol (4.77) represent the major constituents of the oil.

### 2.2. Cytotoxicity of LG and Citral

**Figure 2:**
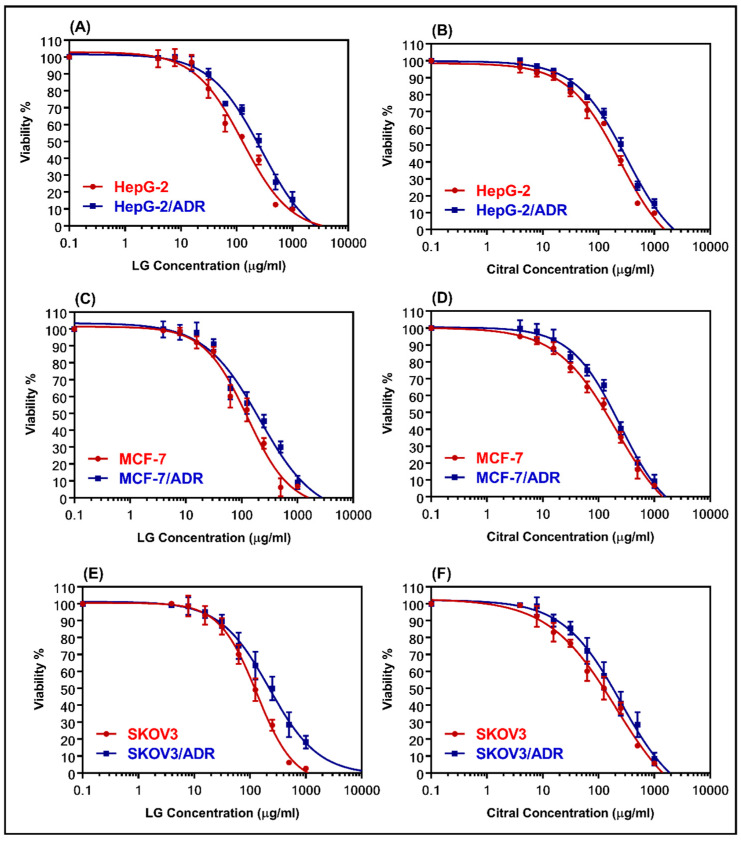
The dose-response curves for LG and citral were determined in sensitive and resistant hepatic (**A**,**B**), breast (**C**,**D**), and ovarian (**E**,**F**) cell lines, respectively. The individual cell lines were incubated for 24 h with several concentrations (0.1–1000 μg/mL) of LG essential oil and the major constituent, citral. The cell proliferation was evaluated using an MTT assay.

The cytotoxicity of LG and citral was determined using MTT assay in both sensitive and resistant cell lines (MCF-7 and MCF-7/ADR, HepG-2, and HepG-2/ADR, and SKOV-3 and SKOV-3/ADR). Figure 2 shows dose–response curves for the samples that were evaluated. Table 2 summarizes the IC_50_ values. LG was more cytotoxic than citral against all the examined cell lines. Table 2 shows that HepG-2/ADR cells are the most resistant to treatment with LG (281.8 µg/mL) and citral (323.3 µg/mL), with relative resistance values of 2.17 and 1.33, respectively. The resistant cells showed high RR values of 7.93, 11.71, and 10.1 for HepG-2/ADR, MCF-7/ADR, and SKOV-3/ADR compared to the sensitive cells, respectively (Table 2).

### 2.3. Combination of LG and Citral with DOX

**Figure 3:**
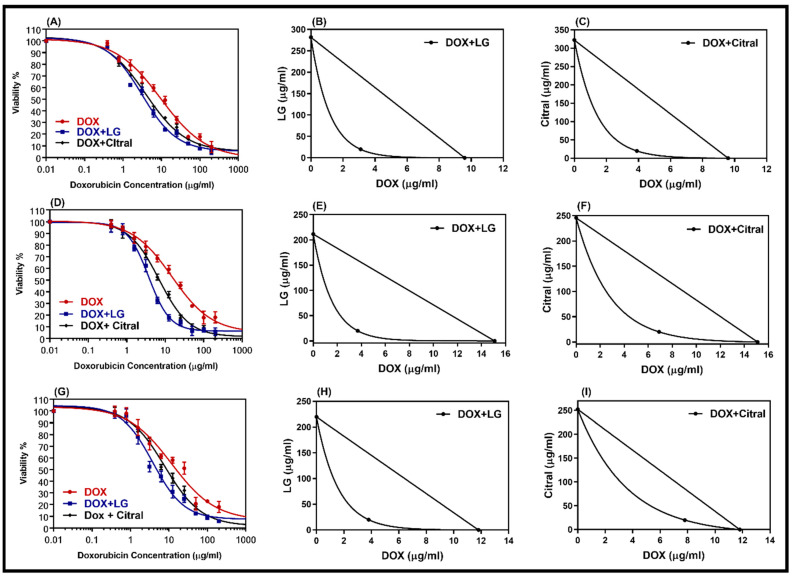
Dose–response curves of doxorubicin alone and in combination with LG or citral in resistance cell lines HepG-2/ADR (**A**), MCF-7/ADR (**D**), and SKOV3/ADR (**G**). The isobologram of DOX with LG (**B**,**E**,**H**) and of DOX with citral (**C**,**F**,**I**) showed a synergism interaction in the tested cells, respectively. Individual cell lines were incubated for 24 h with several concentrations (0.01–200 μg/mL) of DOX with a non-toxic concentration of 20 μg/mL of either LG essential oil or the major constituent, citral. The cell proliferation was evaluated using an MTT assay.

DOX-resistant cell lines were chosen for this study so that we could analyze the reversal effect of the double combination of DOX with LG or citral. The non-toxic concentrations of LG or citral (20 µg/mL) were combined with DOX to treat DOX-resistant cells. A comparison of the dose–response curves of the combination and isobologram (Figure 3) revealed that LG and citral synergistically increased the cytotoxicity of DOX. Table 3 displays the IC_50_ values of DOX, DOX + LG, and DOX + citral in DOX-resistant cells (HepG2/ADR, MCF-7/ADR, and SKOV-3/ADR). The IC_50_ of DOX was decreased by a factor of 3.1, 4.1, and 4.0 (*p* < 0.001) when combined with LG in resistant cell lines, respectively. Hepatic cancer cells (CI = 0.39) showed the greatest synergistic effect of these combinations, followed by ovarian cancer cells (CI = 0.41) and breast cancer cells (CI = 0.43) (Table 3). Citral was less effective with DOX than LG (Table 3).

### 2.4. Reversal of DOX Resistance by LG and Citral

**Figure 4:**
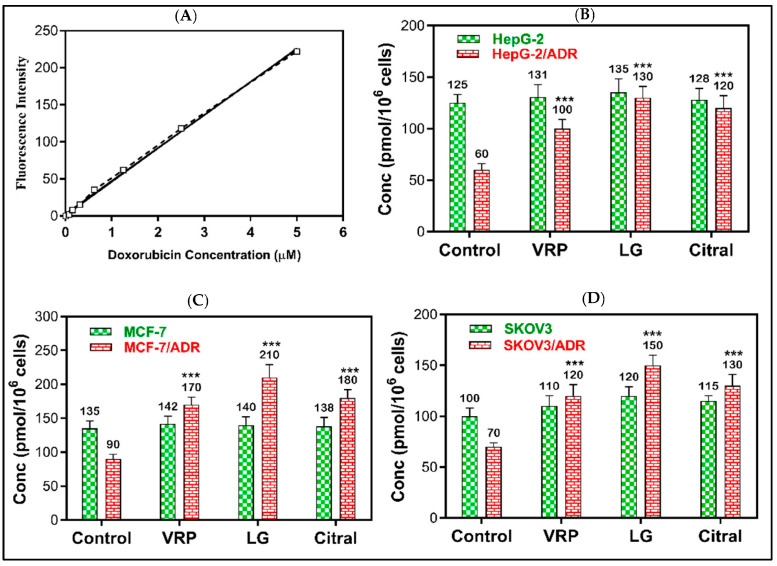
The effect of LG and citral on doxorubicin accumulation in resistant cells. The mean of the fluorescence intensity of DOX plotted against its concentration in the standard curve (**A**), the mean of cellular DOX levels (pmol/106 cells) computed from the standard curve in (**B**) HepG-2 and HepG-2/ADR, (**C**) MCF-7 and MCF-7/ADR, and (**D**) SKOV3 and SKOV3/ADR. *** *p* < 0.001 compared to untreated. Verapamil (10 μM) was used as the positive control.

The reversal effects of DOX + LG and DOX + citral revealed that LG might possess a greater efflux pump (MDR1 and MRP1) inhibitory effect than the positive control verapamil. As demonstrated in Figure 4, 20 μg/mL of LG significantly increased DOX accumulation in HepG-2/ADR, MCF-7/ADR, and SKOV-3/ADR cells by 2.2-, 2.1-, and 2.3-fold, respectively, compared to the untreated cells (*p* < 0.001). Compared to the untreated cells, citral significantly improved DOX retention by 2-, 2-, and 1.9-fold (*p* < 0.001), respectively. However, DOX was non-significantly accumulated in sensitive cells following the treatment with LG or citral.

### 2.5. Modulation of Metabolic Genes by LG and Citral

The RTPCR was used to confirm the resistance development in the MCF-7/ADR, HepG-2/ADR, and SKOV-3/ADR cell lines by evaluating the expression of MDR1. MDR1 expressions were 2.8-fold (*p* < 0.001), 4.6-fold (*p* < 0.001), and 3.7-fold (*p* < 0.001) in resistant cells compared to its expression in parent sensitive cells, respectively.

**Figure 5:**
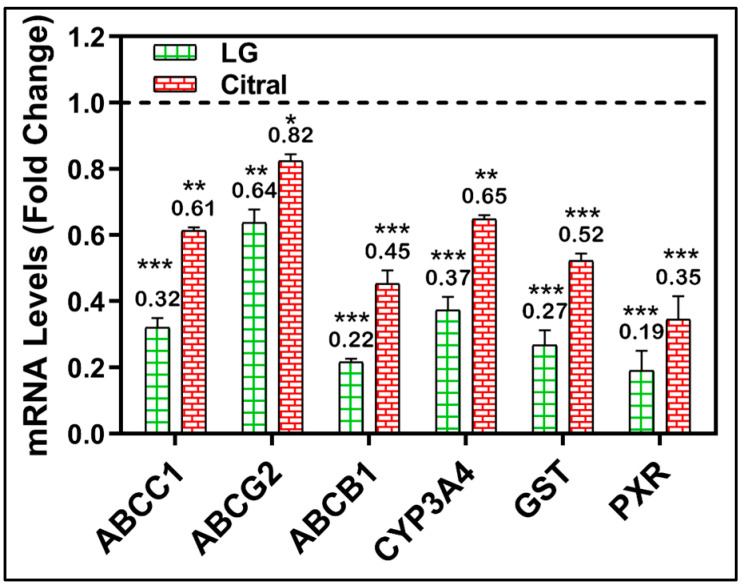
RT-PCR of CYP3A4, GST, PXR, ABCC1, ABCG2, and ABCB1 genes in HepG-2/ADR cells after 24 h of LG, citral therapy. (*n* = 3) Mean ± S.D. * (*p* < 0.05). ** (*p* < 0.01), *** (*p* < 0.001). The data are presented as the mean ± SD of the fold-change related to untreated control and normalized to the β2mg housekeeping gene.

A molecular investigation of metabolically important MDR-related genes was further conducted to validate the modulatory impact of LG and citral. After treating MDR cell lines with LG or citral, the mRNA levels of many MDR-related genes were measured. Figure 5 demonstrates the significant downregulation of the metabolic genes CYP3A4, GST, PXR, ABCC1, ABCG2, and ABCB1 by the treatment with LG and citral (Figure 5).

## 3. Discussion

Although the *C. citraus* is commonly cultivated in Saudi homes, gardens, and streets, consumed as favor drink, and is a part of many folk medicine prescriptions, there are little data about this aromatic herb’s chemical composition and biological activities in Saudi Arabia. Some differences were observed in the essential oil composition compared to other essential oils from other countries. For instance, in plants grown in Egypt, α-citral (34.98%), β-citral (40.72), and myrcene (9.15) dominated the oil [29]. Geranial (39.0%), neral (29.4%), and myrcene (18.0%) also constituted the major composition of the oils from Zambia [30].

Both lemon grass essential oil and citral have been known to have biological effects for a long time. Still, their effects on the drug-resistant phenotype have never been investigated as deeply as in this study. It has been reported that the LG modulated the MDR in bacteria [31,32,33]. However, no intensive study has involved the investigation of MDR on cancer. Here, we revealed that essential oil from lemon grass can modulate multidrug resistance. However, this effect is due to the synergistic interaction of its active constituents and is not limited to its content of citral.

The cytotoxicity of LG ranged from (IC_50_) 126.8 to 131.8 μg/mL for sensitive cells, while resistant cell IC_50_ values ranged from 211.5 to 281.8 μg/mL. Citral had a less cytotoxic effect on sensitive and resistant cells than DPX or LG (Table 1). It has been reported that LC and its active components, including citral, geraniol, geranyl acetate, bisabolol, and iso-intermedeol, have cytotoxic effects on cancer cells [34,35]. The major component of lemon grass oil, citral, may be anti-proliferative against several cancer cell lines, including LNCaP and PC-3 prostate cancer cells, HL60, U937 ovarian cancer cells, cervical cancer cells, MCF-7 breast cancer cells, but not normal epithelial cells [36]. The LG and citral mechanism of killing cancer cells include processes that limit cell migration, the cell cycle, and DNA synthesis, which ultimately result in apoptosis [36].

Combining DOX with LG and citral re-sensitized MDR cells and enhanced the cytotoxic effect of DOX by decreasing the IC_50_ values by >3-fold and >1.5-fold after the combination with LG and citral, respectively. The isobologram and CI indicated the synergistic effect of these combinations, as shown in Table 1.

The modulatory effect of LC and citral on efflux pump function was confirmed by DOX accumulation or reversal assay. Both samples significantly increased the accumulation of DOX inside the resistant cells compared to the untreated cells and verapamil-positive control. Previously, LG and citral treatment significantly reduced cell proliferation and mortality in LU135-wt-src cells compared to LU135-mock cells. LU135-wt-src cells were resistant to standard chemotherapy. SCLC cells were treated with citral and standard chemotherapies. Combination treatment boosted effects on chemo-resistant SCLC cells LU135-wt-src, LU165, and MN1112. Their findings suggested that LG or citral may treat SCLC alone or with chemotherapy [37].

Furthermore, multidrug resistance could be modulated by inhibiting the P-glycoprotein efflux pump. Consequently, the natural mixture of chemicals found in lemon grass essential oil has synergistic effects, and its direct use may be preferable over its usage as a template for citral separation [31].

Individual components have frequently been demonstrated to be less effective than essential oil, probably because the combination of numerous components leads to the synergism of the activity of each molecule. It was discovered that the effects of LG on doxorubicin-resistant ovarian cancer cells were independent of citral [38]. The observed effects, including the modulation of multidrug resistance and the suppression of the P-glycoprotein efflux pump in colon cancer [39] and ovarian carcinoma cells [40], are attributed to the natural variety of bioactive compounds that are present in LG. In addition, additional investigations demonstrated the anticancer impact of LG, despite the inability to attribute anticancer action to its ingredients [41].

RT-PCR confirmed the targeting effect of LG and citral in metabolic molecules. The LG and citral significantly downregulated PXR, CYP3A4, GST, MDR1, MRP1, and PCRP in resistant cell lines (Figure 5). The metabolic phases 1 and 2 are bordered by the drug transporter phases 0 and 3, representing intracellular cytoplasmic drug traffic outside the cells [42].

**Figure 6:**
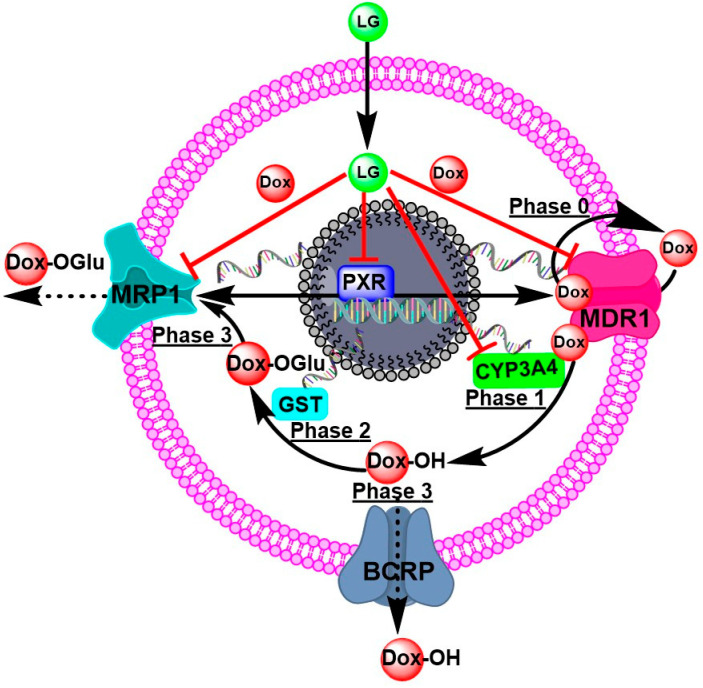
The proposed mechanisms of DOX-resistance and the targeting molecules of LG to overcome DOX-resistance Phase 0 is when P-gp/ABCB1/MDR1 takes up and effluxes the drug. Phases 1 and 2 are biotransformations, such as hydroxylation by CYP3A4 and conjugation by GST. Phase 3 is the transport of DOX-metabolites to the outside of cells for excretion.

P-gp/ABCB1/MDR1 transporters are responsible for phase zero of drug kinetics on cells (phase 0). Phase 0 is the initial stage of DOX uptake. Nevertheless, some molecules of DOX efflux the extracellular space by consuming ATP from the transporters. Certain DOX molecules undergoing Phase 1 hydroxylation by CYP3A4 combine to form a more polar DOX derivative. Phase 2 of metabolism is governed by GST’s conjugation of glutathione with polar molecules. In Phase 3 of metabolism, this polar derivative of DOX is removed by ABC transporters, particularly MRP1. These processes are responsible for reducing DOX’s effectiveness against cancer cells. LG and citral can modulate the nuclear receptor superfamily, the pregnane X receptor (PXR), which regulates the expression of metabolic enzymes and transporters involved in MDR cells’ response to DOX, improving the cytotoxicity of DOX (Figure 6).

## 4. Materials and Methods

### 4.1. Chemicals and Reagents

The RPMI1640 and DMEM medium with the supplemental nutrients were from Gibco^®^ (Thermo Fisher Scientific, Waltham, MA, USA). Sigma-Aldrich^®^ (Taufkirchen, Germany) was used to purchase citral (99%), DOX (98%), verapamil (98%), and “3-(4,5-dimethylthiazol-2-yl)-2,5-diphenyl-tetrazolium bromide” MTT. Applied Biosystems provided their RNeasy Mini Kit, First Strand cDNA Synthesis Kit for PCR, and Master SYBR Green Kit (Los Angeles, CA, USA). VWR^®^ was used to acquire DMSO, methanol (p.a.), and HPLC-grade water (Darmstadt, Germany).

### 4.2. Extraction of LC Essential Oils

*Cymbopogon citratus* (DC) *Stapf*.; lemon grass (LG) was collected in spring and gratefully recognized after being gathered in the Umm Al-Qura University, Abdia, Makkah, Saudi Arabia gardens. A voucher specimen (00121 C) was deposited in the herbarium at Umm Al-Qura University’s faculty of pharmacy’s Pharmacognosy Museum. A total of 700 g of fresh leaves were treated to a three-hour steam distillation in a Clevenger-type device. The oil was dried with calcium chloride and kept in a sealed vial at 4 °C. Based on the fresh herb, the essential oil yield was estimated (1.2%).

### 4.3. GC/MS Analysis

A Shimadzu GC-2010 plus gas chromatograph (Shimadzu Corporation, Kyoto, Japan) coupled with a quadrupole mass spectrometer Shimadzu QP-2010 in addition to an Rtx-5MS fused bonded column (30 m × 0.25 mm i.d. × 0.25 m film thickness) (Restek, Bellefonte, PA, USA) and a split–splitless injector were used for GC/MS analysis. The initial column temperature was 45 °C, which stayed isothermal for 2 min before being set to increase by 5 °C per minute where it remained isothermal for 5 min. The detector temperature was 300 °C, whereas the injector temperature was 250 °C with a 2 mL/min flow rate. Helium was utilized as the carrier gas. For the recording of mass spectra, the filament emission current was 60 mA, the ionization voltage was 70 eV, and the ion source temperature was 200 °C. The diluted samples were injected at 1:15 as the split mode was employed. Using an AOC-20i autosampler, 1 μL of the sample was automatically injected into the chromatograph. The chromatograms were recorded and integrated using GC solution^®^ software version 2.4. (Shimadzu Corporation, Kyoto, Japan). The constituents of the essential oil were determined by comparing retention indices and mass spectra to those previously registered in libraries (NIST Mass Spectral Library (December 2005), Wiley Registry of Mass Spectral Data, 8th edition), the database in this laboratory, and the literature [43,44,45].

### 4.4. Cell Lines

Human cell lines of breast cancer (MCF-7) were grown in complete DMEM media, while liver cancer (HepG-2) and ovarian cancer (SKOV-3) were grown in complete RPMI1640 media under standard conditions at 5% CO_2_, 37 °C, and without mycoplasma. MCF-7/ADR, HepG-2/ADR, and SKOV-3/ADR cell lines resistant to DOX (Adriamycin) were modified by treating and keeping the cells alive in media with 5 µg/mL DOX for 12 weeks to achieve resistance. Comparing the expression of P-gp/ABCB1/MDR1 in DOX-sensitive parent cell lines to RT-PCR, we could prove that DOX resistance had developed in the cell lines. Before any experiments were performed, DOX-free media were set up for 7–10 days.

### 4.5. Cytotoxicity LG and Citral and Their Combination with DOX

In the MTT cell viability experiment, exponentially developing cells (2 × 10^3^ cells/well) were put in 96-well plates [46]. After 24 h of growth, the cells were treated with increasing doses of tested samples (up to 500 µg/mL), DOX (200 µg/mL), and MTT solution (0.5 mg/mL) for 4 h. Formazan crystals, the reaction result, were dissolved in DMSO. At 570 nm, the absorbance was measured with a SpectraMax M5e Multi-Mode Microplate Reader (Molecular Devices, LLC, Los Angeles, CA, USA). The cytotoxicity of the combination of DOX and LG or citral (20 µg/mL) was evaluated using the same methodology.

### 4.6. Efflux Pump Functional Assay

Using the fluorescent P-gp/ABCB1/MDR1 substrate doxorubicin (DOX), we examined the effect of LG and citral on the function of P-gp/ABCB1/MDR1 and MRP efflux pump in resistant cells [47]. As a control, verapamil, an inhibitor of efflux pump was utilized. MCF-7/ADR, HepG-2/ADR, and SKOV-3/ADR cells were grown at a density of 1 × 10^5^ cells per well in 6-well plates. LG and citral (20 μg/mL) were applied for 24 h, then incubated at 37 °C for 30 min with ten μM DOX in 2 mL PBS at 37 °C. The cells were centrifuged after being washed with 2 mL of lysis buffer. Fluorescence intensity was measured at excitation/emission wavelengths of 470 nm/560 nm using a SpectraMax M5e fluorescence spectrometer to assess the intercellular DOX content in cell supernatant. DOX is a substrate for fluorescence for P-gp/ABCB1/MDR1 and MRP1. DOX-resistant cells released DOX, whereas LG and citral-treated cells could accumulate DOX within the cells. The intensity of DOX fluorescence was plotted versus its standard concentrations (standard curve). The amounts of accumulated DOX in resistant and sensitive cells treated with LG and citral were determined and compared to verapamil-treated cells (positive control).

### 4.7. mRNA Levels Using RT-PCR

After 24 h of cultivation, 5 × 10^4^ resistant cells per well were treated with LG, citral, and their combination for 48 h. According to the manufacturer’s instructions, mRNA was isolated using an RNA isolation kit (Applied Biosystem, Waltham, MA, USA). The RNA concentration and quality were determined using a Genova Nano Micro-volume Life Science and Standard Spectrophotometer, and RNA was kept at −80 °C. According to the Reverse Transcription System, 1 mg of RNA, oligo(dT)16 primers, nucleotides, and transcriptase enzyme were used to synthesize cDNA (Promega Corporation; Madison, WI, USA). In a volume of 20 µL, 5 µL of cDNA (1:10) and 0.5 µM of each primer (Table 4) were mixed with 10 µL of Master SYBR Green I (Applied Biosystems, Los Angeles, CA, USA). Gene expressions were identified using Fast RT-PCR 7500 (Applied Biosystem, Los Angeles, CA, USA) with a fast SYBR green experimental setting, and fold changes were calculated according to the 2^−ΔΔCt^ method. The expression of all target genes was normalized in relation to the housekeeping genes β2mg.

### 4.8. Statistical Analysis

Each experiment was conducted three times in triplicate. All data were presented as mean ± standard deviation. The IC_50_ results were computed and shown via GraphPad Prism^®^ (Version 9, GraphPad Software Inc., San Diego, CA, USA). Student’s *t*-test was used to examine the significance among the results, and *p*-values < 0.05 were considered significant.

The following equation was used to compute the relative resistance (RR) of the samples tested:

(1) $$\mathrm{RR}=\frac{{\mathrm{IC}}_{50}\;\mathrm{value}\;\mathrm{obtained}\;\mathrm{for}\;\mathrm{the}\;\mathrm{resistant}\;\mathrm{cell}\;\mathrm{line}\;}{{\mathrm{IC}}_{50}\;\mathrm{value}\;\mathrm{obtained}\;\mathrm{for}\;\mathrm{the}\;\mathrm{sensitive}\;\mathrm{parental}\;\mathrm{cell}\;\mathrm{line}}$$

Fold reversal (FR) for tested samples was calculated using the following equation:

(2) $$\mathrm{FR}=\frac{{\mathrm{IC}}_{50}\;\mathrm{value}\;\mathrm{of}\;\mathrm{DOX}\;\mathrm{on}\;\mathrm{the}\;\mathrm{resistant}\;\mathrm{cell}\;\mathrm{line}\;}{{\mathrm{IC}}_{50}\;\mathrm{value}\;\mathrm{DOX}+\mathrm{LGon}\;\mathrm{the}\;\mathrm{resistant}\;\mathrm{cell}\;\mathrm{line}}$$

Combination index (CI) is the nature of the interaction between DOX and LG and citral (synergy, additivity, or antagonism) was evaluated using the combination index (CI) [54]:

(3) $$\mathrm{CI}=\frac{C_{\mathrm{DOX},50}}{{\mathrm{IC}}_{50,\mathrm{DOX}}}+\frac{C_{\mathrm{LG},50}}{{\mathrm{IC}}_{50,\mathrm{LG}}}$$

where C_DOX,50_ is the cytotoxic agent’s IC_50_ value in a two-drug combination, and C_LG,50_ is the fixed concentration of an LG or citral. IC_50,DOX,_ and IC_50,LG_ are the IC_50_ values for DOX and LG individually. CI < 1 denotes synergism, CI = 1 denotes additive, and CI > 1 denotes antagonism, and the isobologram approach validated the synergism [55].

## 5. Conclusions

This study demonstrates that *C. citratus* and its major constituent, citral, can potentially reverse MDR in cancer cells via modulating ABC transporters and drug metabolism enzymes. The current observations may open new avenues of utilizing *C. citratus* and citral as an adjuvant therapy to prevent the development of multidrug resistance in cancer cells and enhance the treatment of MDR cancer. As a step towards therapeutic application, additional in vivo experiments are required to investigate the modulatory effects of *C. citratus* and citral and their combinations with DOX in rats. 

## Figures and Tables

**Table 1:** Volatile constituents of *C. citrates*.

No.	Compounds	RI	Relative Abundance (%)
1	*β*-Myrcene	987	0.6
2	*β*-Linalool	1103	0.87
3	*β*-Citral	1248	10.15
4	Geraniol	1260	1.21
5	*α*-Citral	1279	18.50
6	Anethole	1287	1.39
7	γ-Terpinen-7-al	1292	0.86
8	Carvacrol	1307	0.94
9	*p*-Mentha-1,4-dien-7-ol	1332	0.6
10	Nerolic acid	1338	1.87
11	*δ*-Elemene	1346	5.38
12	Eugenol	1370	4.77
13	Ylangene	1375	5.70
14	Geranyl acetate	1382	9.65
15	*trans-α*-Bergamotene	1415	2.43
16	*β*-Caryophyllene	1432	0.6
17	*trans-α*-Ionone	1436	1.1
18	*cis-β*-Copaene	1441	2.3
19	*τ*-Gurjunene	1476	2.02
20	Germacrene D	1496	1.82
21	*δ*-Guaiene	1514	0.96
22	Guaiacylacetone	1545	2.88
23	*α*-Calacorene	1552	0.94
24	Caryophylene oxide	1596	1.51
25	Humulane-1,6-dien-3-ol	1610	0.76
26	*δ*-Cadinol	1630	3.09
27	*τ*-Cadinol	1656	1.19
28	*τ*-Muurolol	1669	2.43
29	Eudesm-7(11)-en-4-ol	1676	1.17
30	Farnesal	1737	1.22
31	Myristic acid	1780	0.63
32	Hexahydrofarnesyl acetate	1841	1.48
33	Oleic acid	2161	2.68
34	1-Docosene	2196	0.81
35	(Z)-Methyl communate	2235	0.78
Monoterpene hydrocarbons			0.6
Oxygen-containing monoterpenes			53.69
Sesquiterpene hydrocarbons			19.19
Oxygen-containing sesquiterpenes			13.09
Others			8.72
Total identified			95.29

**Table 2:** IC_50_ values (µg/mL) of the cytotoxicity assay of sensitive and resistant cell lines.

Cells	LG	Citral	DOX
HepG-2	129.7 ± 11.4	242.9 ± 22.1	1.21 ± 0.11
HepG-2/ADR	281.8 ± 16.1 ***	323.3 ± 31.2 **	9.6 ± 0.75 ***
RR	2.17	1.33	7.93
MCF-7	126.8 ± 10.9	210.2 ± 17.1	1.29 ± 0.12
MCF-7/ADR	211.5 ± 17.3 ***	245.8 ± 15.9 *	15.1 ± 1.3 ***
RR	1.67	1.16	11.71
SOVK-3	131.8 ± 9.5	208.4 ±	1.17 ± 0.11
SOVK-3/ADR	219.7 ± 19.7 ***	252.3 ± 23.2 *	11.8 ± 1.2 ***
RR	1.67	1.2	10.1

RR; relative ratio. The * for *p* < 0.05, ** for *p* < 0.01, and *** *p* < 0.001.

**Table 3:** Synergistic interaction of a combination of DOX with LG and citral (20 µg/mL) in HepG-2/ADR-, MCF-7/ADR-, and SOVK-3/ADR-resistant cell lines.

Cells	Combination	IC_50_	FR	CI	r	IB
HepG-2/ADR	DOX	9.6 ± 0.75				
	DOX + LG	3.11 ± 0.29 ***	3.1	0.39	0.99	Synergism
	DOX + Citral	3.94 ± 0.32 ***	2.4	0.47	0.98	Synergism
MCF-7/ADR	DOX	15.1 ± 1.3				
	DOX + LG	3.71 ± 0.24 ***	4.1	0.43	0.98	Synergism
	DOX + Citral	6.86 ± 0.49 ***	2.2	0.54	0.99	Synergism
SOVK-3/ADR	DOX	11.8 ± 1.2				
	DOX + LG	3.81 ± 0.27 ***	4.0	0.41	0.99	Synergism
	DOX + Citral	7.8 ± 0.54 ***	1.5	0.74	0.99	Synergism

CI; combination index, FR; fold reversal, IB; isobologram, and medium effect equation (r-value). *** for *p* < 0.001 comparing to IC_50_ values of DOX.

**Table 4:** Primers used for real-time qPCR.

Gene	Accession	Forward Primer 5′–3′	Reverse Primer 5′–3′	Design
P-gp/ABCB1/MDR1	NM_001348946.1 GI: 1149123048	CCCATCATTGCAATAGCAGG	TGTTCAAACTTCTGCTCCTGA	[48]
MRP1/ABCC1	NM_004996.3 GI: 134142336	ATGTCACGTGGAATACCAGC	GAAGACTGAACTCCCTTCCT	[49]
BCRP/ABCG2	NM_004827.2 GI: 62526032	AGATGGGTTTCCAAGCGTTCAT	CCAGTCCCAGTACGACTGTGACA	[50]
GST	M99422.1 GI: 183662	TACCTGGGCAAGAAGCACGG	AGAGCCCAGAGCAGGTCGTTG	[51]
CYP3A4	NM_017460.5 GI: 322960990	CTAGCACATCATTTGGACTG	ACAGAGCTTTGTGGGACT	[52]
hPXR	NM_003889.3 GI: 148536875	TGTCATGACATGTGAAGGATG	TTGAAATGGGAGAAGGTAGTG	[52]
β2mg	X07621.1 GI: 29298	CCAGCAGAGAATGGAAAGTC	CATGTCTCGATCCCACTTAAC	[53]

## Data Availability

Not applicable.

## Sample Availability

Essential oil of LG and citral samples included in this study available from the authors.
